# Participant-Centric Initiatives and Medical Research: Scoping Review Protocol

**DOI:** 10.2196/resprot.7407

**Published:** 2017-12-12

**Authors:** Victoria Coathup, Nao Hamakawa, Teresa Finlay, Jessica Bell, Jane Kaye, Kazuto Kato

**Affiliations:** ^1^ Centre for Health, Law and Emerging Technologies Nuffield Department of Population Health University of Oxford Oxford United Kingdom; ^2^ Department of Social Medicine Graduate School of Medicine Osaka University Osaka Japan

**Keywords:** patient engagement, patient public involvement, digital technology, patient participation, research participant, dynamic consent, data sharing, patient empowerment

## Abstract

**Background:**

Significant advances in digital technologies have meant that health care data can be collected, stored, transferred, and analyzed for research purposes more easily than ever before. Participant-centric initiatives (PCI) are defined as “tools, programs, and projects that empower participants to engage in the research process” using digital technologies and have the potential to provide a number of benefits to both participants and researchers, including the promotion of public trust in medical research, improved quality of research, increased recruitment and retention, and improved health care delivery.

**Objective:**

The main objective of this scoping review is to describe the extent and range of PCIs across the United Kingdom, United States, and Japan that are designed to facilitate medical research.

**Methods:**

The methodological framework described by Levac et al will be applied to this scoping review. We will search electronic databases (MEDLINE, EMBASE, PsychINFO, Cumulative Index to Nursing, and Allied Health Literature and CiNii), grey literature sources, Internet search engines (Google and Bing), and hand search key journals and reference lists of relevant articles. All digital tools and programs will be eligible for inclusion if there is a description of key features and functions that fall within the parameters of a PCI. Only those that play a role in medical research will be included.

**Results:**

Preliminary searches conducted in MEDLINE and EMBASE retrieved 1820 and 2322 results, respectively. The scoping review will be completed by January 2018.

**Conclusions:**

The scoping review will be the first to map the extent and range of PCIs currently available across the United Kingdom, United States, and Japan, and will be the first review to contribute to a better understanding of what PCIs patients may benefit from. Researchers and practitioners will be able to use information in this review as a guide for patients and also as a guide for the development of future tools and programs. The results will be disseminated through a peer-reviewed publication and conference presentations.

## Introduction

Over the last few decades there have been significant advances in digital technologies for health care provision and medical research. These offer new and innovative ways to recruit participants and conduct research, including the use of biobanks, data repositories, and social media, as well as the potential for secondary analyses of routinely collected data, such as administrative data and medical records [1,2]. This technological progress also means data can be stored, transferred, and analyzed more easily than ever before.

While there is vast potential for this to improve health care, there is a growing concern for participants’ privacy, in particular how their data is being used and who has access to it. A traditional model of participant consent applies to the majority of research that is conducted; therefore, it is possible that once broad consent has been obtained, unbeknown to participants, their data may be shared with a variety of actors [3]. These technical advances and improved capabilities have coincided with the recognition of the importance of involving the public more closely in medical research, especially with regard to their views on privacy and consent.

The traditionally paternalistic attitude to medical research is also changing, with a shift towards a participant-centered model where the individual takes a more “active” role in the research process. It is believed that this approach is more ethical, can improve the quality of the research conducted, and may enhance the agency and control individuals have over their health and relevant data [4]. While the evidence to support this is currently limited, there remains a growing consensus about the importance of adopting this approach [5].

Participant-centric initiatives (PCIs) are platforms and programs that have been developed in order to facilitate the “active” role participants can take in medical research. PCIs are defined as “tools, programs, and projects that empower participants to engage in the research process” using digital technologies [6]. The characteristics can vary greatly between PCIs, however, the core features remain the same: the participant is placed at the center of decision-making and the user interface enables them to engage with the research process. Detailed explanations of PCIs and their features have been published elsewhere [6,7]. Briefly, PCIs fall into the following broad categories: (1) matchmaking, (2) direct-to-consumer (DTC), (3) dynamic negotiation, and (4) citizen science.

The matchmaking category refers to digital tools and platforms that enable individuals to connect and communicate with researchers and identify studies in which they might be eligible to participate, based on their personal information and preferences. Matchmaking tools put the individual in control of when, how, and what types of studies they are invited to participate in.

The DTC category refers to the commercial organizations that offer individuals a service in addition to opportunities to communicate with researchers and ways of being involved in research. Services can include social media networks where individuals can interact with others who have the same condition. Evidence suggests that peer-to-peer support has a variety of benefits for patients, particularly for those who are suffering from rare or stigmatizing conditions [8,9]. Other services may include genetic testing or tools to search for opportunities to participate in clinical trials.

Tools that were developed to give individuals greater control of how their data is used and shared for research purposes fall into the dynamic negotiation category. This offers participants an alternative to the traditional broad consent model that usually accompanies participation in medical research, enabling them to tailor their preferences and expectations [3].

Citizen science is a form of research driven by participants where they are heavily involved in some or all of the design, data collection, analysis, and dissemination of the study findings. A number of platforms have been designed to encourage this type of community-based approach to research that engages and involves participants at various points throughout the process. This type of research means that citizens can direct the research agenda to their own interests, develop their knowledge and skill sets, and bring a range of perspectives and expertise to a project [10].

PCIs have the potential to provide a number of benefits to both participants and researchers, including the promotion of public trust in medical research, improved quality of research, increased recruitment and retention, and improved health care delivery [7]. Because of this, we believe it is important to understand what PCIs are currently available to researchers and potential participants, and how they differ in terms of their key features and functions.

The main objective of this review is to describe the variety and prevalence of PCIs across 3 countries: United Kingdom, United States, and Japan.

We chose these countries because they differ with regard to the health care systems in place, the levels of engagement and involvement patients typically have in decisions regarding medical care and research participation, and public attitudes towards participation in medical research [11-14].

The United Kingdom and United States are leaders at the forefront of the patient-centric approach to health care. National organizations, such as INVOLVE and *Patient-Centered Outcomes Research Institute* (PCORI), which were set up over the past decade, provide support and guidance to stakeholders regarding the involvement and engagement of patients in medical research [15], and enable more informed health care decision-making through that research [16].

In comparison, in Japan, a paternalistic model of health care largely remains and research indicates that there is widespread satisfaction in this model [17]. A qualitative study conducted in Japan (2004) indicated that members of the public often make decisions about participating in research studies based on whether they trust the doctor they are speaking to or not, and described feelings of obligation when asked to participate by a doctor they like and trust. Participants also expressed a lack of interest in medical research; they felt it was something disassociated from them [14].

A survey conducted in 2009 by the National Institute of Science and Technology Policy (NISTEP) of the Ministry of Education, Culture, Sports, Science, and Technology (MEXT) in Japan suggested that members of the general population are less interested in issues regarding science compared with individuals in the United Kingdom and United States [18]. However, a study we conducted in Japan that explored attitudes of patients to the potential use of digital technology for engaging with health care and medical research revealed that patients are interested in the use of digital platforms for this purpose [19]. So there is growing support in Japan for a more patient-centered approach to health care and medical research [17,20], but it is still very much in its infancy.

These differences in infrastructure and culture make for interesting comparisons in the types of PCIs that are currently available in the United Kingdom, United States, and Japan.

## Methods

A scoping review was considered the most appropriate design to address the aims of this study for a number of reasons. Firstly, the aims of this review are very broad and unlike a systematic review or meta-analysis, we are not trying to answer a specific question, but rather “examine the extent, range, and nature of a research activity” [21]. Secondly, a scoping review is rigorous and requires implementing a comprehensive and systematic approach to searching for relevant literature. The scoping review methodological framework described by Levac et al [22] will be applied to this scoping review. The framework is based on the seminal work of Arksey and O’Malley [21] and comprises the following stages: (1) identifying the research question; (2) identifying relevant studies; (3) study selection; (4) charting the data; (5) collating, summarizing, and reporting the results; and (6) consultation.

### Stage 1: Identifying the Research Question

This study has built on the work by Kaye et al [7], which discussed the key features, benefits, and challenges of implementing PCIs. We describe examples of PCIs currently available for research purposes. Understanding the range and features of PCIs available was deemed important as it could inform researchers’, clinicians’, and also potential research participants’ decisions about research. The research question was developed by a multidisciplinary research team which included academic researchers from a range of fields including law, bioethics, and public health, and was refined as the research team became more familiar with the literature.

#### Key Definitions

We use the term “medical research” throughout to refer to a broad range of research that can be applied to medical treatments, services, and settings. In addition, we refer to the “extent and range” of PCIs throughout this protocol. By “extent” we are referring to the prevalence of PCIs within each country and “range” refers to the type of PCI (using the categories described above) and the key features of each PCI included in the study.

#### Research Question

Our research question is: What is the extent and range of participant-centric initiatives available for medical research across the United Kingdom, United States, and Japan?

#### Objectives

The objectives of the study are to (1) identify existing PCIs currently used for medical research purposes across the United Kingdom, United States, and Japan; (2) compare the number and types of PCIs available within the United Kingdom, United States, and Japan; (3) estimate the number of participants using such platforms, tools, and programs across each country; (4) where possible, identify the model of consent used; (5) identify and describe the key features of PCIs available within each country; and (6) identify gaps in PCI provision within the United Kingdom, United States, and Japan.

### Stage 2: Identifying Relevant Studies

To guide the search strategy a set of parameters was developed by the research team, which included inclusion and exclusion criteria (Textbox 1), which scientific databases to search, where to search for grey literature, search terms to use, search limitations applied, and which experts to consult with regard to review findings. To verify which countries a particular PCI is available in, we will take the following multistage approach: (1) identify the country of origin or use reported either in the journal article describing the PCI or directly on the website, platform, or tool; (2) if it is not possible to identify this from the journal article or PCI website, platform, or tool, we will contact the organization that developed the PCI or the authors of the journal article; (3) if we do not receive a response detailing the country of origin/use, we will try to identify the Web address of any Internet-based PCI (ie, ‘.co.uk’ or ‘.jp’) and any country-specific requirements for participants to access PCIs (ie, National Health Service [NHS] number in the United Kingdom or social security number in the United States). In the event that we identify PCIs that are based in one country but available for use in another, we will report the PCI as available in both countries. For example, some PCIs are based in the United States, but open to a global audience, such as Patients Like Me. This would be reported twice, as a PCI that is available within both the United Kingdom and United States. Because the aims of this review are broad, we felt it was beyond the remit of this review to include all citizen science platforms, programs, or tools. The term “citizen science” is used to describe a wide variety of activities [10] and we have decided to take a pragmatic approach and focus our search on matchmaking, dynamic negotiation, and DTC PCIs only.

### Search Strategy

Three systematic reviews assessing different aspects of patient and public involvement (PPI) within health care decisions [23-25] were used to develop the original search strategy for this scoping review. Preliminary searches were conducted in MEDLINE and EMBASE to further develop the search terms, Medical Subject Headings (MeSH), and limitations used. A specialist subject librarian was also consulted and provided guidance on search strategy.

**Inclusion and exclusion criteria.**

Inclusion

- Article or website describing PCI
- Published in English language or Japanese
- Adult population (18 or more years)
- Focus on medical/health care research purposes
- Complies with PCI definition described by Anderson et al [6]
  - Digital device or tool/computer program/digital platform
  - Enables potential participant to take initiative within either of the following research processes:
    - To connect and communicate with researchers
    - To control what data is used and shared for research purposes
  - Available to participants residing in the United Kingdom, United States, or Japan

Exclusion

- Platforms that enable patients to connect and communicate with other patients only
- Platforms that use data for research, but participants are not engaged or empowered by the process
- Citizen science

Search terms included a mixture of keywords and MeSH terms using combinations of the terms listed in Textbox 2. Proximity search functions were used to link related terms and narrow the search. Similar searches will also be conducted in Cumulative Index to Nursing and Allied Health Literature (CINAHL) and Psych-Info. Equivalent terms in Japanese will be used to search CiNii.

Due to the topic of this scoping review, it is likely that many PCIs will exist that are not written about in scientific literature. Therefore, we will conduct a thorough search of grey literature to identify non-indexed relevant literature. The grey literature search will focus on conference abstracts and PPI organization websites. We will therefore search Open Grey (grey literature database), Google, and Bing using the same terms listed in Textbox 2.

We will also hand search key journals (Digital Health, The Journal of mHealth, International Journal of Digital Health care, and Journal of Japan Society for Health care Management), and the reference lists of relevant articles for citations that were not identified from the original database search.

Finally, we will contact experts within the field of PCIs and invite them to participate as part of an expert panel. The panel will be made up of bioethicists, clinicians, PPI experts, and digital health specialists who reside in the United Kingdom, United States, and Japan. The panel will be consulted to provide feedback on our findings and to ensure that we have identified all relevant literature.

All searches will be conducted by members of the research team and reference management software will be used to store all relevant literature.

### Stage 3: Study Selection

The study selection process will be conducted in 2 stages. In the first stage, one researcher in the United Kingdom will conduct the search for English language literature and another researcher will conduct the search for Japanese literature. Titles and abstracts or website content will be reviewed based on the defined inclusion and exclusion criteria. At this initial stage the goal is to be more inclusive and articles or websites will only be excluded if it is clear that they fall outside of the eligibility criteria. If the reviewer is uncertain at this stage, the article or website will be included. In the second stage, full text articles will be obtained and 2 reviewers will independently review the articles and websites that have been collected in the first stage. The articles and websites will be grouped into 3 categories: included, excluded, and uncertain. The reviewers will then compare categories to ensure inter-rater reliability and validity. If there are any discrepancies that cannot be resolved, a meeting will be held with a third reviewer to discuss the articles and websites until a consensus is reached. If a consensus cannot be reached, the decision of the majority will be taken.

### Stage 4: Charting the Data

This stage involves extracting the relevant data from included articles and websites that will help to address the original aims of the scoping review. A data extraction form will be developed based on key characteristics by the research team and members of an expert panel will be consulted to ensure that all relevant details will be obtained.

Two researchers will then pilot the data extraction form on the first 25 articles or websites to be included. The research team will meet to discuss findings of the pilot to decide whether amendments need to be made.

**Search terms.**

- Participant
  - participant
  - public
  - citizen
  - stakeholder
  - communit*
- Engagement
  - engage*
  - involve*
  - PPI
  - citizen science
  - participatory
  - empower
  - consult
  - partner
  - collaborate
  - collaboratory
  - crowdsourc*
- Medical research
  - trial
  - RCT
  - research
  - studies
  - study
  - qualitative
  - quantitative
  - evaluation
  - observational
  - cohort
  - case control
  - research design
- Technology
  - technology
  - digital
  - platform
  - online
  - Internet
  - computer
  - website
  - software
  - program

**Preliminary steps in the data extraction process.**

- Researcher performing data extraction and date conducted
- Identifying characteristics of article or website (ie, author, year, title, website name, organization)
- Country of origin/use (United Kingdom, United States, or Japan)
- Description of PCI (aim, type of platform, and method of engagement)
- Approximate number of users
- Participant requirements for use such as subscription or access fee, clinician referral, participant personal identifier (ie, NHS number, social security number, post/zip code, etc)
- Type of organization (private/public or for profit/not for profit)
  Target population (general population or specific patient groups, global, or country specific)
- Nature of research conducted
  - Participant invited to take part in primary research studies
  - Secondary use of data already available (ie, routinely collected hospital data from medical records or data generated from discussion between participants)
- Who will conduct the research/access data
  - Commercial organizations
  - Public sector/non-profit researchers
  - Universities/other educational institutes
  - Other
- Type of interaction
  - Matchmaking
  - Dynamic negotiation
  - Direct-to-consumer
- Stakeholder interaction
  - Participant to participant
  - Participant to researcher
  - Participant to clinician/health care professional
- Model of consent
  - Broad consent: a participant signs up and agrees their data can be used for any research relevant
  - Explicit consent: a participant’s consent is requested before each use with a study
  - Dynamic consent: a participant can specify particular future uses of their data that they will allow and uses that they will not allow
- Any additional key features
  - Ability to withdraw
  - Control over level of contact (study invitations/follow up procedures)
  - Other

This is an iterative process and a number of versions will be developed and reviewed before the full data extraction process can be conducted. Data extraction will be completed by 2 researchers and a random sample of articles will be chosen for review by a third researcher to ensure validity. Preliminary steps are shown in Textbox 3.

### Stage 5: Collating, Summarizing and Reporting the Results

The breadth of this scoping review means it is likely that a large amount of data will be generated. In order to adequately address the aims of this scoping review, which are to map the extent and range of PCIs available across the United Kingdom, United States, and Japan, the results will be presented in the following ways: (1) we will use the Preferred Reporting Items for Systematic Reviews and Meta-Analysis (PRISMA) flow chart template reporting the search process [26]; (2) results tables presenting the key characteristics of included PCIs that will be stratified by country and potentially also by patient groups; and (3) a narrative analysis which will include a description of key findings, a critical analysis of included PCIs, and summary of gaps within PCI provision across the 3 countries of interest.

The findings will be presented in accordance with the PRISMA reporting guidelines where appropriate. We will also consult our expert panel members after a first draft of results has been developed to explore whether our search strategy has identified all key PCIs that are related to our research objectives.

### Stage 6: Consultation With Expert Panel

The consultation stage of the scoping review will provide opportunities for input from a variety of stakeholders to ensure that the knowledge produced from the project is relevant and accessible. We will employ an expert panel of stakeholders who we will consult with during stages 4 and 5 to ensure we are gathering data that is considered important, that our search strategy has identified all relevant PCIs, and that our findings are clear and understandable. The expert panel will be comprised of researchers within the area of PPI, patient engagement and health care digital technologies, clinicians, and members of patient organizations (lay patient/public representatives). In addition to this, the final stage of this review will ensure that the knowledge generated as part of this study will be disseminated to all relevant stakeholders. A lay summary of the findings will be produced and reviewed by our patient/public representatives. The final version will be sent to members of the wider stakeholder community along with a full text article of the review.

## Results

Preliminary searches were conducted in November 2016. Initially, searches were retrieving too many results, rendering it impractical. Advice from a specialist librarian was sought and the search strategy was refined by combining search terms that described the study population and characteristics of PCI (ie, participant/ patient/ public AND engagement/ involvement/ recruitment, etc). The subsequent searches were conducted in MEDLINE and EMBASE and retrieved 1820 and 2322 results, respectively.

The remaining stages of the proposed scoping review will be complete in January 2018.

## Discussion

The aim of this scoping review is to map the extent and range of PCIs currently available across the United Kingdom, United States, and Japan. This will be the first scoping review conducted within this area and will contribute to a better understanding of what PCIs patients may benefit from. The rapid advances in digital technologies over the last decade have contributed to a shift in how a lot of medical research is approached, including the ability to link large administrative datasets, which has led to an increase in the analysis of routinely collected data for medical research purposes [27,28]. However, with these benefits also comes an increase in privacy concerns for the participants within these research studies. PCIs have the potential to facilitate research recruitment, increase retention rates, and build public trust with researchers by transferring more control to participants and providing a more transparent view of the research process. Therefore, by identifying gaps within the current market, we hope that new and innovative platforms will be developed to engage, empower, and involve potential participants within the research process.

## Abbreviations

DTC: direct-to-consumer
PCI: participant-centric initiatives
PPI: patient and public involvement
PRISMA: Preferred Reporting Items for Systematic Reviews and Meta-Analysis
